# Same duodenal neuroendocrine tumors, different endoscopic resection methods: a case report and literature review

**DOI:** 10.3389/fmed.2024.1401241

**Published:** 2024-06-04

**Authors:** Jinguo Liu, Liangliang Yu

**Affiliations:** ^1^Department of Endoscopy Center, Sir Run Run Shaw Hospital, Zhejiang University, Hangzhou, China; ^2^Department of Gastroenterology, The Second Affiliated Hospital, Zhejiang Chinese Medical University, Hangzhou, China

**Keywords:** neuroendocrine tumor, duodenal bulb, endoscopic resection, ESD, STER

## Abstract

Duodenal neuroendocrine tumors (NETs), comprising 2–3% of all gastrointestinal NETs and 1–3% of all duodenal tumors, are remarkably uncommon. In this report, we described a patient diagnosed with two submucosal tumors in the duodenal bulb. We used two distinct endoscopic resection methods, including endoscopic submucosal dissection (ESD) and submucosal tunneling endoscopic resection (STER), to achieve en bloc resection of the lesions without complications. Pathological evaluation, involving hematoxylin–eosin staining and immunohistochemistry, confirmed the diagnosis of NET. Given the limited operative field and space in the duodenal bulb, STER proved to be a viable endoscopic resection technique.

## Introduction

Neuroendocrine tumors (NETs), distinguished by their ability to secrete various biogenic amines and peptide hormones, are heterogeneous neoplasms derived from the secretory cells of the diffuse neuroendocrine system (1). Predominantly found in the gastroenteropancreatic tract and bronchopulmonary tree, duodenal NETs are particularly rare (1), accounting for only 2–3% of all gastrointestinal NETs and 1–3% of all duodenal tumors (2). However, possibly due to heightened awareness, the early detection of these tumors has increased, largely attributable to the widespread use of endoscopy and imaging techniques (3). Over recent decades, the incidence of NETs has increased. Data from the Surveillance, Epidemiology, and End Results (SEER) 18 registry indicate that between 2000 and 2012, the highest incidence rates of gastrointestinal NETs reached 3.56 per 100,000 persons, with a higher prevalence in males (4).

The biological aggressiveness of NETs varies considerably depending on their primary site, with NETs originating in the small bowel, including the duodenum, typically exhibiting a higher malignant potential, despite their relatively indolent progression in metastatic stages (1, 5). Notably, at the time of diagnosis, 40–60% of duodenal NETs have already metastasized to regional lymph nodes (3). However, duodenal NETs are often detected incidentally, as they usually do not present with specific clinical symptoms (6).

Surgery is the primary treatment for localized NETs that are symptomatic, of intermediate-to-high grade, or larger than 2 cm, particularly in the case of pancreatic NETs. However, the management of non-functioning, low-grade, small (≤ 2 cm), incidentally detected NETs remains a subject of debate. Additional treatment modalities include immunotherapy, peptide receptor radioligand therapy, somatostatin analog therapy, symptomatic management, and endoscopic resection. In China, the methods of endoscopic resection, including endoscopic submucosal excavation (ESE) (7), endoscopic submucosal dissection (ESD) (8), and submucosal tunneling endoscopic resection (STER) (9) are increasingly favored for treating these smaller and less invasive NETs, due to their reduced risk of complications. Moreover, endoscopic resection serves as an intermediate strategy that bridges the gap between a “watch-and-wait” approach and surgery according to the horizontal and vertical margins.

## Case description

**Figure 1:**
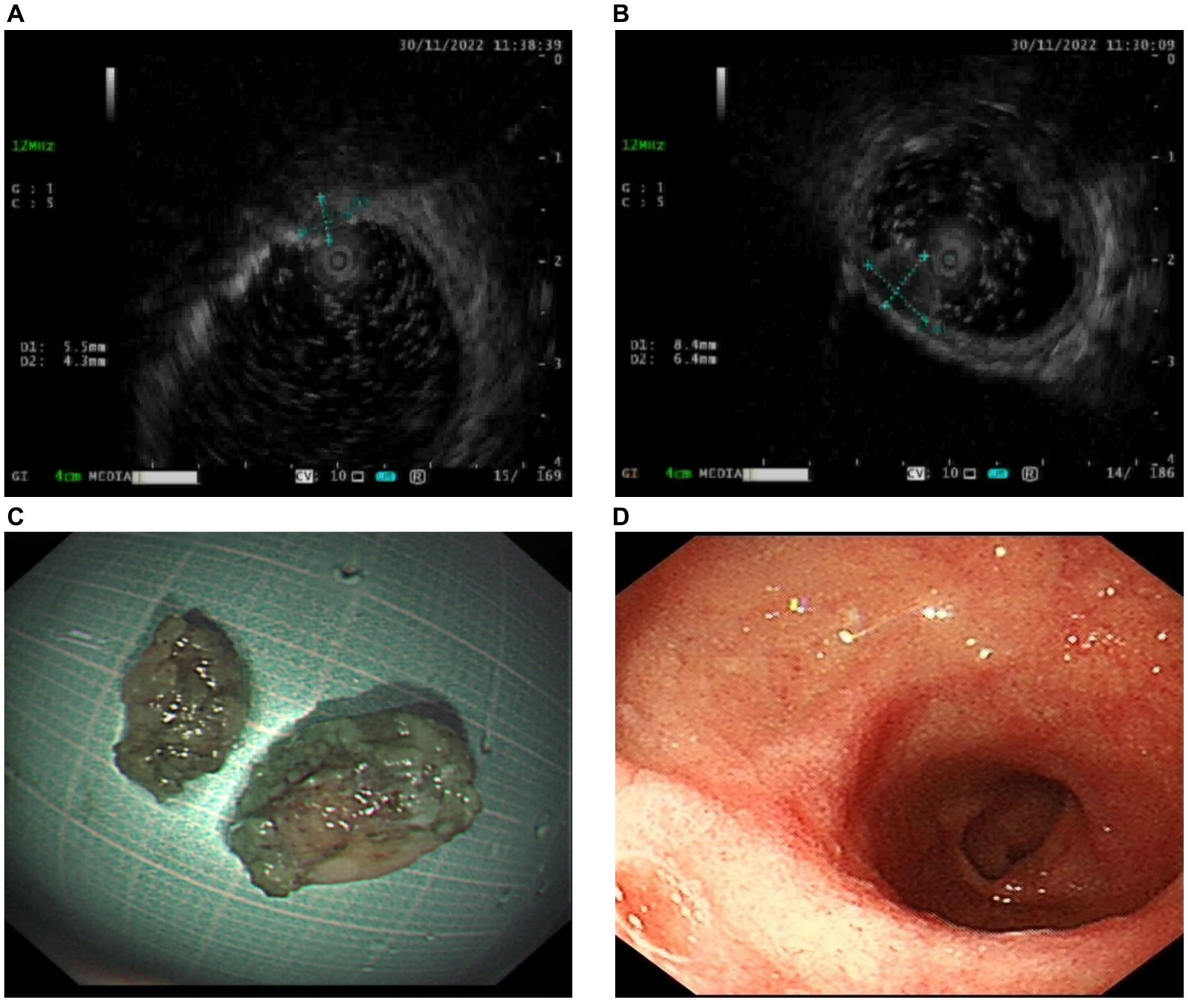
Endoscopic ultrasonography and resected lesions. **(A,B)** Endoscopic ultrasonography shows two hypoechoic lesions (**A** measuring 5.5*4.3 mm and **B** measuring 8.4*6.4 mm) originating from muscularis propria of the duodenal bulb. **(C)** The resected lesions were pulled out. **(D)** Postoperative gastroscopy 3 months later showed the healing of the resection sites.

A 59-year-old woman was referred to our hospital following the discovery of two submucosal tumors (SMTs) in the duodenal bulb during a routine gastroscopy. She was asymptomatic with no significant findings on physical examination and laboratory tests. Endoscopic ultrasonography revealed two hypoechoic lesions originating from the muscularis propria of the duodenal bulb (Figures 1A,B).

**Figure 2:**
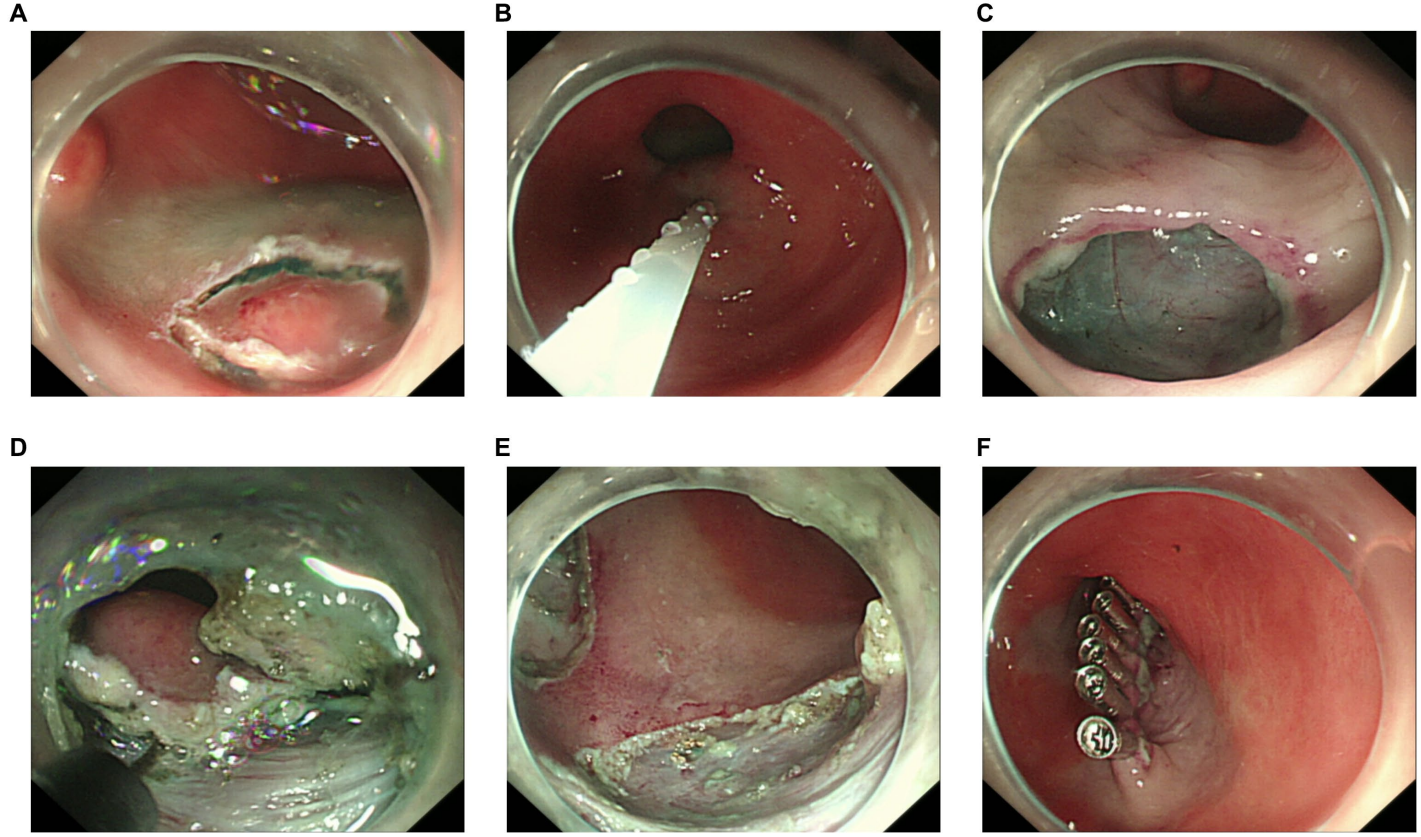
Procedure of submucosal tunneling endoscopic resection. **(A)** The lesion behind the pyloric ring. **(B)** Submucosal injection of sodium hyaluronate, methylene blue, and glycerol fructose solution (1:1:4). **(C)** Submucosal tunnel incision. **(D)** Submucosal tunnel to the lesion. **(E)** En bloc resection. **(F)** Tunnel closure with titanium clips.

**Figure 3:**
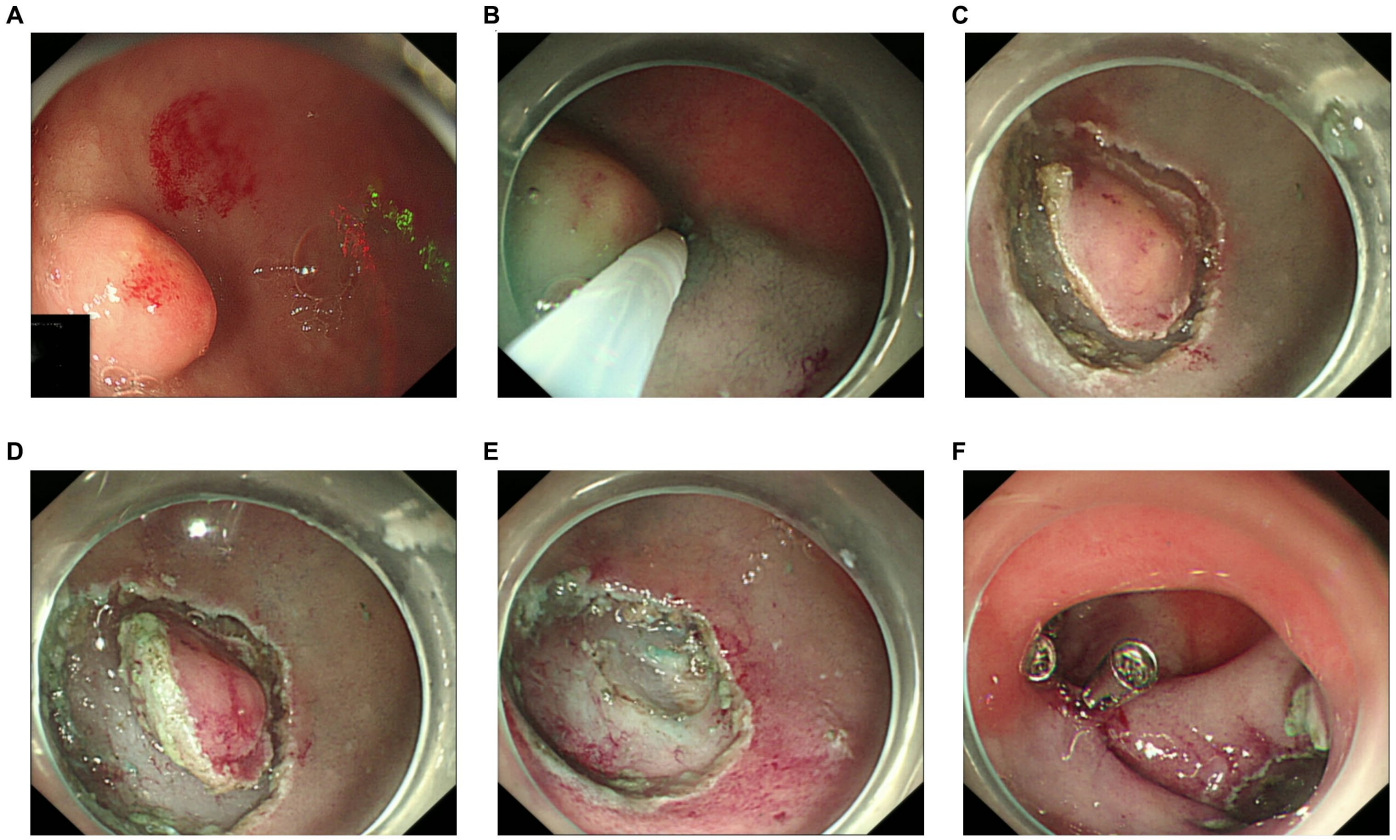
Procedure of endoscopic submucosal dissection. **(A)** The lesion in the anterior wall. **(B)** Submucosal injection. **(C)** A circumferential mucosal incision. **(D)** Submucosal dissection. **(E)** En bloc resection. **(F)** Titanium clips close the wound.

**Figure 4:**
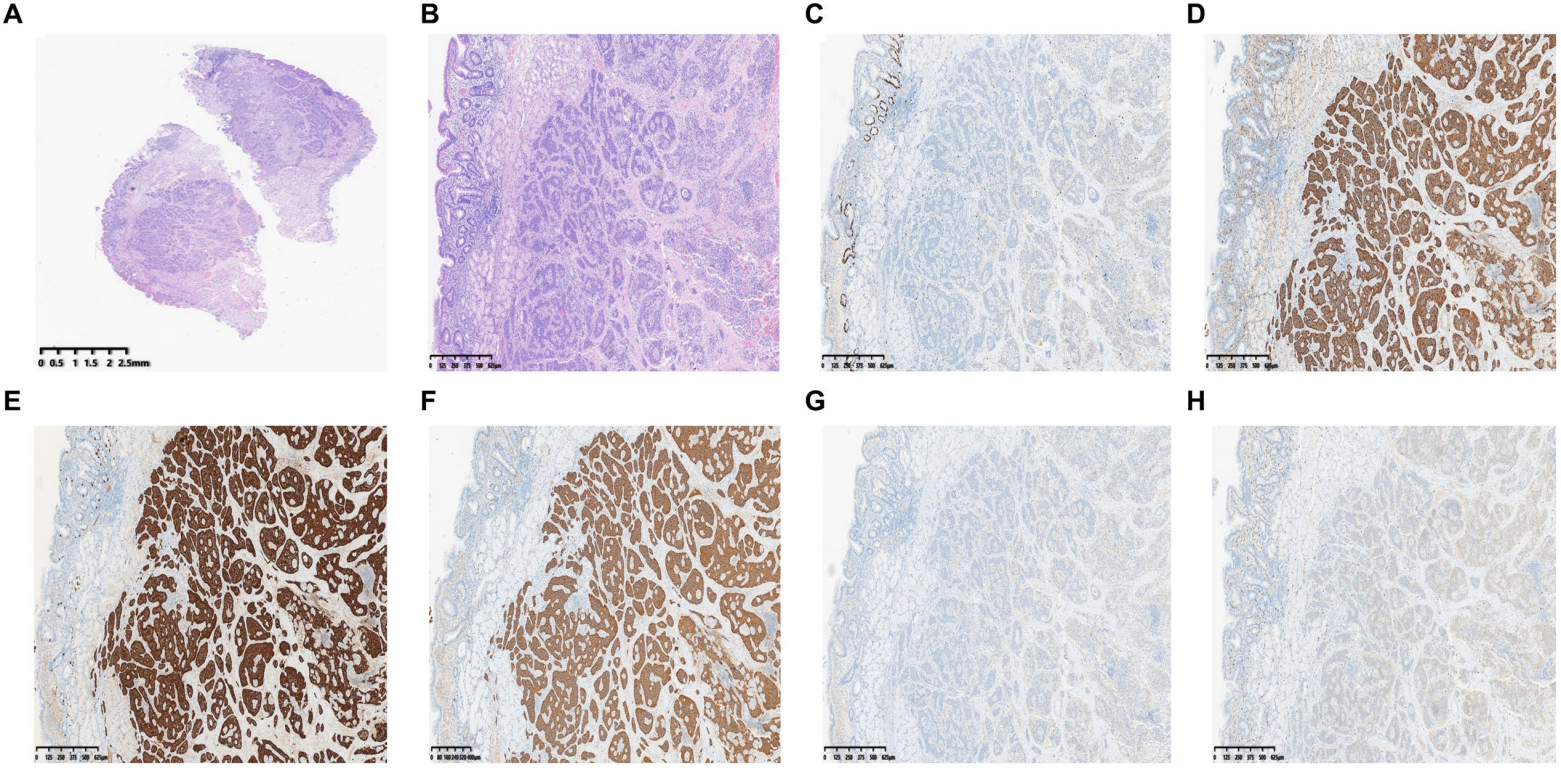
Pathologic examination. **(A)** Gross images. **(B)** Hematoxylin–eosin. **(C)** Immunohistochemistry shows positive areas for Ki-67 (< 3%). **(D–F)** Immunohistochemistry shows positive areas for CD56 **(D)**, CgA **(E)**, and synaptophysin **(F)**. **(G, H)** Immunohistochemistry of somatostatin **(G)** and gastrin **(H)**.

Because an SMT in the greater curvature side was hidden behind the pyloric ring, it had poor exposure to the visual field under endoscopy and great difficulty in ESD. It was finally resected with the method of STER (Figure 2). Another one in the anterior wall was removed by the method of ESD (Figure 3). The completely resected SMTs were pulled out (Figure 1C). Pathologic examination (Figure 4A) by hematoxylin–eosin staining (Figure 4B) and immunohistochemistry (Figures 4C–F) that were positive for Ki-67(< 3%), the cluster of differentiation 56 (CD56), chromogranin A (CgA), and synaptophysin were both consistent with a NET. Moreover, immunohistochemistry results of somatostatin (Figure 4G) and gastrin (Figure 4H) were negative, which eliminated the suspicion of somatostatinomatosis and gastrinoma. Three months later, the patient underwent a follow-up gastroscopy that showed complete wound healing (Figure 1D).

## Discussion

The diagnosis of NETs in the duodenal bulb is particularly challenging considering the absence of characteristic neuroendocrine syndromes and the rarity of this malignancy. Additionally, the anatomical constraints of the duodenum, including obstruction by the pyloric ring, a thin muscularis propria, and limited operative field and space, complicate the use of minimally invasive endoscopic techniques (10, 11). These challenges contribute to a lower rate of en bloc resection and an increased risk of complications during the resection of duodenal bulb lesions (12, 13).

ESD stands out as a highly effective technique for treating non-ampullary duodenal neoplasms with its high rate of en bloc resection and low recurrence rate (14, 15). Despite these advantages, the method is associated with significant risks, including bleeding and perforation, which pose substantial clinical challenges. Notably, several studies have reported high rates of delayed perforation (14.3–28.6%) and bleeding (0–22%) when using ESD for duodenal lesions (16, 17). Furthermore, the anatomical constraints of locations such as the posterior wall of the duodenal bulb or areas behind the pyloric ring complicate the procedure. These factors not only make en bloc resection more challenging but also heighten the risk of complications. This underscores the need for advanced techniques or modified approaches to enhance safety and efficacy in these complex scenarios.

STER uses submucosal tunneling to facilitate en bloc resection, effectively addressing the anatomical challenges posed by traditional ESD. This technique proves particularly advantageous for lesions located on the posterior wall of the duodenal bulb or behind the pyloric ring, where conventional ESD may falter. Despite its efficacy, the potential for submucosal tunneling injuries should not be overlooked. Luo et al. (9) reported a 100% success rate in en bloc resection for lesions in the duodenal bulb via STER, with no significant bleeding and only a minor micro-perforation, approximately 3 mm in width. However, these findings, while promising, underscore the necessity for larger clinical studies to validate the safety and effectiveness of STER in broader patient populations.

Endoscopic resection is increasingly favored over surgery for duodenal NETs, occasionally pushing the boundaries of tumor size eligibility. However, the optimal management of these cases remains ambiguous. The prognosis for duodenal NETs varies considerably, with up to 50% of patients presenting with metastatic disease at initial diagnosis, particularly in cases involving functioning neoplasms (18). Given this high variability and risk, a rigorous local staging process, incorporating nuclear imaging or endoscopic ultrasound, is strongly recommended prior to undertaking endoscopic resection (18, 19). This approach ensures a more accurate assessment of the tumors’ extent and feasibility for endoscopic management.

## Data availability statement

The original contributions presented in the study are included in the article/supplementary material, further inquiries can be directed to the corresponding author.

## Ethics statement

Written informed consent was obtained from the individual(s) for the publication of any potentially identifiable images or data included in this article.

## Author contributions

JL: Writing – original draft, Writing – review & editing. LY: Conceptualization, Validation, Writing – review & editing.
