# Corrigendum: Same duodenal neuroendocrine tumors, different endoscopic resection methods: a case report and literature review

**DOI:** 10.3389/fmed.2025.1577088

**Published:** 2025-02-27

**Authors:** Jinguo Liu, Liangliang Yu

**Affiliations:** ^1^Department of Endoscopy Center, Sir Run Run Shaw Hospital, Zhejiang University, Hangzhou, China; ^2^Department of Gastroenterology, The Second Affiliated Hospital, Zhejiang Chinese Medical University, Hangzhou, China

**Keywords:** neuroendocrine tumor, duodenal bulb, endoscopic resection, ESD, STER

In the published article, there was an error regarding the affiliation(s) for [Jinguo Liu]. As well as having affiliation(s) [1], they should also have [2].

The authors apologize for this error and state that this does not change the scientific conclusions of the article in any way. The original article has been updated.

